# The DIRECT consortium and the REST-meta-MDD project: towards neuroimaging biomarkers of major depressive disorder

**DOI:** 10.1093/psyrad/kkac005

**Published:** 2022-06-09

**Authors:** Xiao Chen, Bin Lu, Hui-Xian Li, Xue-Ying Li, Yu-Wei Wang, Francisco Xavier Castellanos, Li-Ping Cao, Ning-Xuan Chen, Wei Chen, Yu-Qi Cheng, Shi-Xian Cui, Zhao-Yu Deng, Yi-Ru Fang, Qi-Yong Gong, Wen-Bin Guo, Zheng-Jia-Yi Hu, Li Kuang, Bao-Juan Li, Le Li, Tao Li, Tao Lian, Yi-Fan Liao, Yan-Song Liu, Zhe-Ning Liu, Jian-Ping Lu, Qing-Hua Luo, Hua-Qing Meng, Dai-Hui Peng, Jiang Qiu, Yue-Di Shen, Tian-Mei Si, Yan-Qing Tang, Chuan-Yue Wang, Fei Wang, Hua-Ning Wang, Kai Wang, Xiang Wang, Ying Wang, Zi-Han Wang, Xiao-Ping Wu, Chun-Ming Xie, Guang-Rong Xie, Peng Xie, Xiu-Feng Xu, Hong Yang, Jian Yang, Shu-Qiao Yao, Yong-Qiang Yu, Yong-Gui Yuan, Ke-Rang Zhang, Wei Zhang, Zhi-Jun Zhang, Jun-Juan Zhu, Xi-Nian Zuo, Jing-Ping Zhao, Yu-Feng Zang, the DIRECT consortium, Chao-Gan Yan, Xiao Chen, Li-Ping Cao, Wei Chen, Yu-Qi Cheng, Yi-Ru Fang, Qi-Yong Gong, Wen-Bin Guo, Li Kuang, Bao-Juan Li, Tao Li, Yan-Song Liu, Zhe-Ning Liu, Jian-Ping Lu, Qing-Hua Luo, Hua-Qing Meng, Dai-Hui Peng, Jiang Qiu, Yue-Di Shen, Tian-Mei Si, Yan-Qing Tang, Chuan-Yue Wang, Fei Wang, Hua-Ning Wang, Kai Wang, Xiang Wang, Ying Wang, Xiao-Ping Wu, Chun-Ming Xie, Guang-Rong Xie, Peng Xie, Xiu-Feng Xu, Hong Yang, Jian Yang, Shu-Qiao Yao, Yong-Qiang Yu, Yong-Gui Yuan, Ke-Rang Zhang, Wei Zhang, Zhi-Jun Zhang, Jun-Juan Zhu, Xi-Nian Zuo, Jing-Ping Zhao, Yu-Feng Zang, Chao-Gan Yan

**Affiliations:** CAS Key Laboratory of Behavioral Science, Institute of Psychology, Chinese Academy of Sciences, Beijing 100101, China; International Big-Data Center for Depression Research, Chinese Academy of Sciences, Beijing 100101, China; Magnetic Resonance Imaging Research Center, Institute of Psychology, Chinese Academy of Sciences, Beijing 100101, China; Department of Psychology, University of Chinese Academy of Sciences, Beijing 100049, China; International Big-Data Center for Depression Research, Chinese Academy of Sciences, Beijing 100101, China; Magnetic Resonance Imaging Research Center, Institute of Psychology, Chinese Academy of Sciences, Beijing 100101, China; International Big-Data Center for Depression Research, Chinese Academy of Sciences, Beijing 100101, China; Magnetic Resonance Imaging Research Center, Institute of Psychology, Chinese Academy of Sciences, Beijing 100101, China; CAS Key Laboratory of Behavioral Science, Institute of Psychology, Chinese Academy of Sciences, Beijing 100101, China; International Big-Data Center for Depression Research, Chinese Academy of Sciences, Beijing 100101, China; Magnetic Resonance Imaging Research Center, Institute of Psychology, Chinese Academy of Sciences, Beijing 100101, China; Department of Psychology, University of Chinese Academy of Sciences, Beijing 100049, China; Sino-Danish College, University of Chinese Academy of Sciences, Beijing 101408, China; Sino-Danish Center for Education and Research, Graduate University of Chinese Academy of Sciences, Beijing 101408, China; International Big-Data Center for Depression Research, Chinese Academy of Sciences, Beijing 100101, China; Magnetic Resonance Imaging Research Center, Institute of Psychology, Chinese Academy of Sciences, Beijing 100101, China; Department of Child and Adolescent Psychiatry, NYU Grossman School of Medicine, New York, NY 10016, USA; Nathan Kline Institute for Psychiatric Research, Orangeburg, New York, NY 10962, USA; Affiliated Brain Hospital of Guangzhou Medical University, Guangzhou 510370, China; Capital Normal University, Beijing 100089, China; Department of Psychiatry, Sir Run Run Shaw Hospital, Zhejiang University School of Medicine, Hangzhou 310020, Zhejiang, China; Department of Psychiatry, First Affiliated Hospital of Kunming Medical University, Kunming, Yunnan 650032, China; International Big-Data Center for Depression Research, Chinese Academy of Sciences, Beijing 100101, China; Sino-Danish College, University of Chinese Academy of Sciences, Beijing 101408, China; Sino-Danish Center for Education and Research, Graduate University of Chinese Academy of Sciences, Beijing 101408, China; International Big-Data Center for Depression Research, Chinese Academy of Sciences, Beijing 100101, China; Magnetic Resonance Imaging Research Center, Institute of Psychology, Chinese Academy of Sciences, Beijing 100101, China; Shanghai Mental Health Center, Shanghai Jiao Tong University School of Medicine, Shanghai 200030, China; Huaxi MR Research Center (HMRRC), Department of Radiology, West China Hospital of Sichuan University, Chengdu, Sichuan 610044, China; Research Unit of Psychoradiology, Chinese Academy of Medical Sciences, Chengdu, Sichuan 610052, China; Department of Psychiatry, and National Clinical Research Center for Mental Disorders, The Second Xiangya Hospital of Central South University, Changsha 410011, Hunan, China; International Big-Data Center for Depression Research, Chinese Academy of Sciences, Beijing 100101, China; Magnetic Resonance Imaging Research Center, Institute of Psychology, Chinese Academy of Sciences, Beijing 100101, China; Department of Psychiatry, The First Affiliated Hospital of Chongqing Medical University, Chongqing 400042, China; Xijing Hospital of Air Force Military Medical University, Xi'an, Shaanxi 710032, China; Center for Cognitive Science of Language, Beijing Language and Culture University, Beijing 100083, China; Affiliated Mental Health Center & Hangzhou Seventh People's Hospital, Zhejiang University School of Medicine, Hangzhou, Zhejiang 310063, China; Mental Health Center and Psychiatric Laboratory, West China Hospital of Sichuan University, Chengdu, Sichuan 610044, China; CAS Key Laboratory of Behavioral Science, Institute of Psychology, Chinese Academy of Sciences, Beijing 100101, China; International Big-Data Center for Depression Research, Chinese Academy of Sciences, Beijing 100101, China; Magnetic Resonance Imaging Research Center, Institute of Psychology, Chinese Academy of Sciences, Beijing 100101, China; CAS Key Laboratory of Behavioral Science, Institute of Psychology, Chinese Academy of Sciences, Beijing 100101, China; International Big-Data Center for Depression Research, Chinese Academy of Sciences, Beijing 100101, China; Magnetic Resonance Imaging Research Center, Institute of Psychology, Chinese Academy of Sciences, Beijing 100101, China; Department of Clinical Psychology, Suzhou Psychiatric Hospital, The Affiliated Guangji Hospital of Soochow University, Suzhou, Jiangsu 215003, China; Department of Psychiatry, and National Clinical Research Center for Mental Disorders, The Second Xiangya Hospital of Central South University, Changsha 410011, Hunan, China; Shenzhen Kangning Hospital, Shenzhen, Guangzhou 518020, China; Department of Psychiatry, The First Affiliated Hospital of Chongqing Medical University, Chongqing 400042, China; Department of Psychiatry, The First Affiliated Hospital of Chongqing Medical University, Chongqing 400042, China; Shanghai Mental Health Center, Shanghai Jiao Tong University School of Medicine, Shanghai 200030, China; Faculty of Psychology, Southwest University, Chongqing 400715, China; Department of Diagnostics, Affiliated Hospital, Hangzhou Normal University Medical School, Hangzhou, Zhejiang 311121, China; National Clinical Research Center for Mental Disorders (Peking University Sixth Hospital) & Key Laboratory of Mental Health, Ministry of Health (Peking University), Beijing 100191, China; Department of Psychiatry, First Affiliated Hospital, China Medical University, Shenyang, Liaoning 110122, China; Beijing Anding Hospital, Capital Medical University, Beijing 100120, China; Department of Psychiatry, First Affiliated Hospital, China Medical University, Shenyang, Liaoning 110122, China; Early Intervention Unit, Department of Psychiatry, Affiliated Nanjing Brain Hospital, Nanjing Medical University, Nanjing 210024, China; Xijing Hospital of Air Force Military Medical University, Xi'an, Shaanxi 710032, China; Department of Neurology, The First Affiliated Hospital of Anhui Medical University, Hefei, Anhui 230022, China; Department of Psychiatry, and National Clinical Research Center for Mental Disorders, The Second Xiangya Hospital of Central South University, Changsha 410011, Hunan, China; The First Affiliated Hospital of Jinan University, Guangzhou, Guangdong 250024, China; International Big-Data Center for Depression Research, Chinese Academy of Sciences, Beijing 100101, China; Magnetic Resonance Imaging Research Center, Institute of Psychology, Chinese Academy of Sciences, Beijing 100101, China; Xi'an Central Hospital, Xi'an, Shaanxi 710004, China; Department of Neurology, Affiliated ZhongDa Hospital of Southeast University, Nanjing, Jiangsu 210009, China; Department of Psychiatry, and National Clinical Research Center for Mental Disorders, The Second Xiangya Hospital of Central South University, Changsha 410011, Hunan, China; Institute of Neuroscience, Chongqing Medical University, Chongqing 400016, China; Chongqing Key Laboratory of Neurobiology, Chongqing 400000, China; Department of Neurology, The First Affiliated Hospital of Chongqing Medical University, Chongqing 400042, China; Department of Psychiatry, First Affiliated Hospital of Kunming Medical University, Kunming, Yunnan 650032, China; Department of Radiology, The First Affiliated Hospital, College of Medicine, Zhejiang University, Hangzhou, Zhejiang 310058, China; Chongqing Key Laboratory of Neurobiology, Chongqing 400000, China; Department of Psychiatry, and National Clinical Research Center for Mental Disorders, The Second Xiangya Hospital of Central South University, Changsha 410011, Hunan, China; The First Affiliated Hospital of Anhui Medical University, Hefei, Anhui 230032, China; Department of Psychosomatics and Psychiatry, Zhongda Hospital, School of Medicine, Southeast University, Nanjing, Jiangsu 210009, China; First Hospital of Shanxi Medical University, Taiyuan, Shanxi 030001, China; West China Hospital of Sichuan University, Chengdu, Sichuan 610044, China; Department of Neurology, Affiliated ZhongDa Hospital of Southeast University, Nanjing, Jiangsu 210009, China; Department of Psychiatry, Shanghai Jiao Tong University School of Medicine, Shanghai 200025, China; Developmental Population Neuroscience Research Center, IDG/McGovern Institute for Brain Research, Beijing Normal University, Beijing 100091, China; National Basic Science Data Center, Beijing 100038, China; Department of Psychiatry, and National Clinical Research Center for Mental Disorders, The Second Xiangya Hospital of Central South University, Changsha 410011, Hunan, China; Center for Cognition and Brain Disorders, The Affiliated Hospital of Hangzhou Normal University, Hangzhou, Zhejiang 310018, China; Zhejiang Key Laboratory for Research in Assessment of Cognitive Impairments, Hangzhou, Zhejiang 310000, China; CAS Key Laboratory of Behavioral Science, Institute of Psychology, Chinese Academy of Sciences, Beijing 100101, China; International Big-Data Center for Depression Research, Chinese Academy of Sciences, Beijing 100101, China; Magnetic Resonance Imaging Research Center, Institute of Psychology, Chinese Academy of Sciences, Beijing 100101, China; Department of Psychology, University of Chinese Academy of Sciences, Beijing 100049, China; Sino-Danish College, University of Chinese Academy of Sciences, Beijing 101408, China; Sino-Danish Center for Education and Research, Graduate University of Chinese Academy of Sciences, Beijing 101408, China

**Keywords:** major depressive disorder, DIRECT, R-fMRI, database, neuroimaging

## Abstract

Despite a growing neuroimaging literature on the pathophysiology of major depressive disorder (MDD), reproducible findings are lacking, probably reflecting mostly small sample sizes and heterogeneity in analytic approaches. To address these issues, the Depression Imaging REsearch ConsorTium (DIRECT) was launched. The REST-meta-MDD project, pooling 2428 functional brain images processed with a standardized pipeline across all participating sites, has been the first effort from DIRECT. In this review, we present an overview of the motivations, rationale, and principal findings of the studies so far from the REST-meta-MDD project. Findings from the first round of analyses of the pooled repository have included alterations in functional connectivity within the default mode network, in whole-brain topological properties, in dynamic features, and in functional lateralization. These well-powered exploratory observations have also provided the basis for future longitudinal hypothesis-driven research. Following these fruitful explorations, DIRECT has proceeded to its second stage of data sharing that seeks to examine ethnicity in brain alterations in MDD by extending the exclusive Chinese original sample to other ethnic groups through international collaborations. A state-of-the-art, surface-based preprocessing pipeline has also been introduced to improve sensitivity. Functional images from patients with bipolar disorder and schizophrenia will be included to identify shared and unique abnormalities across diagnosis boundaries. In addition, large-scale longitudinal studies targeting brain network alterations following antidepressant treatment, aggregation of diffusion tensor images, and the development of functional magnetic resonance imaging-guided neuromodulation approaches are underway. Through these endeavours, we hope to accelerate the translation of functional neuroimaging findings to clinical use, such as evaluating longitudinal effects of antidepressant medications and developing individualized neuromodulation targets, while building an open repository for the scientific community.

## Introduction

Major depressive disorder (MDD) is the second leading cause of health burden worldwide (Ferrari *et al*., 2013). Unfortunately, objective biomarkers to assist in diagnosis are still lacking, and current first-line treatments are only modestly effective (Borowsky *et al*., 2000; Williams *et al*., 2011), reflecting our incomplete understanding of the pathophysiology of MDD. Characterizing the neurobiological basis of MDD promises to support developing more effective diagnostic approaches and treatments.

An increasingly used approach to reveal neurobiological substrates of clinical conditions is termed resting-state functional magnetic resonance imaging (R-fMRI) (Biswal, 2012). Despite intensive efforts to characterize the pathophysiology of MDD with R-fMRI, clinical imaging markers of diagnosis and predictors of treatment outcomes have yet to be identified. Previous reports have been inconsistent, sometimes contradictory, impeding the endeavour to translate them into clinical practice (Yan *et al*., 2019). One reason for inconsistent results is low statistical power from small sample size studies (Button *et al*., 2013). Low-powered studies are more prone to produce false positive results, reducing the reproducibility of findings in a given field (Ioannidis, 2005; Poldrack *et al*., 2017). Of note, one recent study demonstrated that a sample size of thousands of participants may be needed to identify reproducible brain-wide association findings (Marek *et al*., 2022), calling for larger datasets to boost effect size. Another reason could be the high analytic flexibility (Carp, 2012). Recently, Botvinik-Nezer and colleagues (Botvinik-Nezer *et al*., 2020) demonstrated the divergence in results when independent research teams applied different workflows to process an identical fMRI dataset, highlighting the effects of 'researcher degrees of freedom' [i.e. heterogeneity in (pre-)processing methods] in producing disparate fMRI findings.

To address these critical issues, we initiated the Depression Imaging REsearch ConsorTium (DIRECT) in 2017. Through a series of meetings, a group of 17 participating hospitals in China agreed to establish the first project of the DIRECT initiative, the REST-meta-MDD project, and share 25 study cohorts, including R-fMRI data from 1300 MDD patients and 1128 normal control participants. On the basis of our previous work, a standardized preprocessing pipeline adapted from Data Processing Assistant for Resting-State fMRI (DPARSF) (Yan *et al*., 2016; Yan & Zang, 2010) was implemented at each local participating site to minimize heterogeneity in preprocessing methods. R-fMRI metrics can be vulnerable to physiological confounds such as head motion (Ciric *et al*., 2018; Ciric *et al*., 2017). Based on our previous work examining head motion impact on R-fMRI functional connectivity (FC) connectomes (Yan *et al*., 2013) and other recent benchmarking studies (Ciric *et al*., 2017; Parkes *et al*., 2018), DPARSF implements a regression model (Friston-24 model) on the participant- and group-level correction for mean frame displacements as the default setting.

Participating groups first preprocessed R-fMRI images with a DPARSF standardized protocol at local hospitals, then shared the final R-fMRI indices along with demographic (age, sex, and education) as well as clinical information (first episode/recurrent, medication usage, illness severity, etc.). The REST-meta-MDD project was intended to boost statistical power by pooling functional data across centers, while minimizing the effects of heterogeneous analytical strategies and creating an openly available dataset for the global scientific community. As of 1 January 2020, the dataset of deidentified imaging derivatives was made available for unrestricted sharing. All researchers can obtain access to these R-fMRI indices and corresponding demographic/clinical information via http://rfmri.org/REST-meta-MDD, and perform any analyses of interest without putting participant privacy or confidentiality at risk.

Since its launch, DIRECT has encouraged independent investigations. Data sharing was conducted in two phases. In the initial coordinated sharing phase, all researchers who sought access to the dataset needed to submit a written proposal to the consortium review board. The aims and research design of proposals were evaluated to minimize conflicts with already approved research proposals. The consortium also provided technical support for participating sites regarding preprocessing and statistical analysis as these issues can be challenging for clinical researchers. Through these practices, DIRECT sought to provide a platform that would allow all participating sites to leverage the large R-fMRI database and explore it independently. At the time of writing, DIRECT investigators have published several peer-reviewed research papers. Here, we review the principal findings from these published studies, summarized in Table 1, and discuss the implications of these results and the future directions of DIRECT.

**Table 1:** A summary of DIRECT studies.

Study	Modality	Sample size	Primary results
Yan *et al*., 2019. *Proceedings of the Natational Academy of Sciences USA*	fMRI: network FC	848 MDDs vs. 794 HCs	MDD patients showed significantly reduced FC within DMN (*t* = −3.762, *P* = 0.0002, Cohen's *d* = −0.186) compared to HCs and this effect could only be observed in recurrent MDD patients (*t* = −3.737, *P* = 0.0002, Cohen's *d* = −0.326).
Long *et al*., 2020. *NeuroImage: Clinical*	fMRI: dynamic brain network metrics	460 MDDs vs. 473 HCs	MDD patients showed a higher temporal variability (*F* = 10.218, *P* = 0.000216, FDR corrected), a lower temporal correlation coefficient (*F* = 15.071, *P* = 0.0000333, FDR corrected), and a shorter characteristic temporal path length (*F* = 8.768, *P* = 0.000314, FDR corrected).
Liang *et al*., 2020. *NeuroImage: Clinical*	fMRI: data-driven clustering	690 MDDs vs. 707 HCs	MDD patients could be grouped into two subgroups based on FCs within the DMN with *K*-means clustering method. Subgroup 1’s FC within DMN was enhanced while Subgroup 2’s DMN FC was decreased compared to HCs. The significance of clustering was determined with bootstrapping at the level of *P* < 0.05.
Yang *et al*., 2021. *Molecular Psychiatry*	fMRI: topological feature	821 MDDs vs. 765 HCs	Both global (*E*_glob_: *t* = −2.601, *P* = 0.009) and local efficiency (*E*_loc_: *t* = −2.771, *P* = 0.006) were reduced in MDD patients compared to HCs. Again, this effect was only significant in recurrent MDD patients (*E*_glob_: *t* = −3.893, *P* < 0.001; *E*_loc_: *t* = −4.429, *P* < 0.001).
Liu *et al*., 2021. *Progress in Neuro-Psychopharmacology and Biological Psychiatry*	sMRI: VBM	572 MDDs vs. 481 HCs	Significant differences regarding GMV were found in temporal lobes, fusiform gyrus and thalamus among MDD patients with GI, MDD patients without GI, and HCs.
Ding *et al*., 2021. *Journal of Affective Disorders*	fMRI: PAS	753 MDDs vs. 451 HCs	MDD patients were characterized with increased PAS scores in DMN, FPCN, dorsal, and ventral attention network regions compared to HCs.
Deng *et al*., 2021. *Bipolar Disorder*	fMRI: VMHC	1004 MDDs vs. 898 HCs	Decreased VMHC was found in DMN, VN, and SMN regions in MDD patients compared with HCs.

Abbreviations: VBM, voxel-based morphometry; MDD, major depressive disorder; HC, healthy control; DMN, default mode network; VN, visual network; FPCN, fronto-parietal control network; SMN, somato-motor network; GI, gastrointestinal; PAS, parameter of asymmetry; VMHC, voxel-mirrored homotopic connectivity; FC, functional connectivity; GMV, grey matter volume.

## Principal Findings from DIRECT Studies

### FC abnormalities in MDD

**Figure 1:**
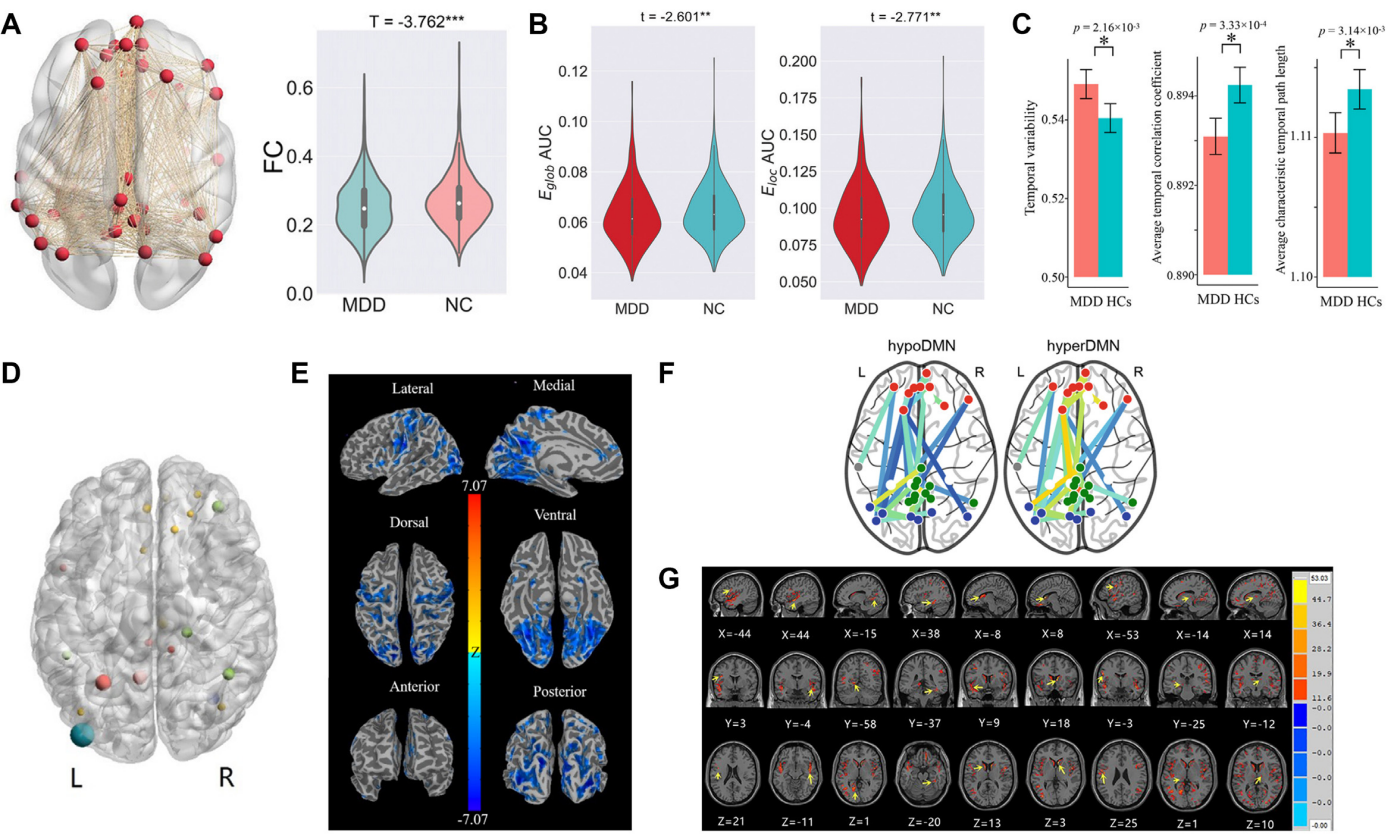
Principal findings from DIRECT studies. (A) Reduced FC within the DMN is revealed in patients with MDD compared to HCs (Yan *et al*., 2019). (B) Both decreased global efficiency (*E*_glob_) and local efficiency (*E*_loc_) are found in MDD vs. HC contrast (Yang *et al*., 2021). (C) Alterations in terms of temporal dynamic properties (increased variability, decreased temporal correlation coefficient, and characteristic temporal path length) are observed in patients with MDD as compared to HCs (Long *et al*., 2020). (D) Altered PAS scores are primarily observed in DMN (red), VN (blue), FPCN (yellow), and ventral and dorsal attention network (green) in MDD vs. HC contrast (Ding *et al*., 2021). (E) Reduced VMHC was found in DMN, VN, and SMN regions in MDD vs. HC contrast (Deng *et al*., 2021). (F) Patients with MDD can be clustered into two subgroups according to FCs within DMN (Liang *et al*., 2020). (G) Temporal and occipital regions, thalamus, prefrontal, and postfrontal gyrus show difference in GMV among GI, non-GI MDD patients, and HCs (Liu *et al*., 2021). Abbreviations: PAS, parameter of asymmetry; GI, gastrointestinal; MDD, major depressive disorder; HC, healthy control; DMN, default mode network; VN, visual network; FPCN, fronto-parietal control network; SMN, somato-motor network; FC, functional connectivity; VMHC, voxel-mirrored homotopic connectivity.

The first DIRECT study (Yan *et al*., 2019) concentrated on a simple but surprisingly controversial theme: FC within the default mode network (DMN) in depression. The DMN was first recognized as a set of brain regions showing reduced haemodynamic activity during externally directed attention tasks and increased activity during resting state or internally focused tasks (Raichle, 2010; Raichle *et al*., 2001; Raichle & Snyder, 2007). By consensus, MDD was considered to be characterized by enhanced FC within the DMN, which was also proposed to be a neural mechanism underlying rumination (Greicius *et al*., 2007; Hamilton *et al*., 2015; Kaiser *et al*., 2015). However, previous findings regarding FC within the DMN in patients with MDD were inconsistent (for a review, see Yan *et al*., 2019). Thus, DIRECT first conducted a mega-analytic investigation, that is, pooling individual-level measures across sites and conducting regression analysis on this pooled dataset. Potentially confounding site effects were corrected with a linear mixed model with a random intercept for sites. Such an analytical approach can boost statistical power to detect subtle effects and allow for flexible control of confounders (Schmaal *et al*., 2020). The mega-analysis also investigated the effects of certain phenotypes: the number of episodes, medication usage, and illness duration. Contrary to initial assumptions, FC in patients with MDD was significantly lower than in HCs within the DMN (*t* = −3.762, *P* = 0.0002). This effect was only observed in patients with recurrent MDD (*t* = −3.737, *P* = 0.0002), and not in first episode drug naïve patients (*t* = −0.914, *P* = 0.361). Overall, MDD patients were found to be characterized by a general yet subtle decrease of FC within the DMN (Fig. 1A). This contradicted some of the previous literature. However, previous studies showing increased DMN FC in MDD were primarily conducted with Caucasian samples, while the sample from REST-meta-MDD was homogeneously Chinese. Ethnic differences in MDD have been consistently reported. Compared with Caucasians, Asians have lower prevalence rates (Ferrari *et al*., 2013), more psychosomatic symptoms (Ryder *et al*., 2008), and different risk genes (Bigdeli *et al*., 2017). Hence, one critical future direction for DIRECT is to identify potential cultural and ethnic differences by pooling cross-cultural samples with international collaborators (see an example of neurodevelopment from Dong *et al*., 2020).

### Topological abnormalities of functional brain networks in MDD

Subsequently, the topological properties of functional brain networks in patients with MDD have been examined (Bullmore & Sporns, 2009; Yang *et al*., 2021) (Fig. 1B). The individual-level R-fMRI data from the REST-meta-MDD project allowed building a topological network with a predefined brain atlas, i.e. Dosenbach's 160 atlas (Dosenbach *et al*., 2010). The effort focused on two essential features of networks, their global (*E*_glob_) and local efficiencies (*E*_loc_) (Rubinov & Sporns, 2010). Both significantly decreased global efficiency (*t* = −2.601, *P* = 0.009) and local efficiency (*t* = −2.771, *P* = 0.006) were found in patients with MDD compared to HCs. Once again, this effect was only significant in patients with recurrent MDD (*E*_glob_: *t* = −3.893, *P* < 0.001; *E*_loc_: *t* = −4.429, *P* < 0.001) and not in first episode drug naïve patients (*E*_glob_: *t* = −0.224, *P* > 0.05; *E*_loc_: *t* = −0.586, *P* > 0.05). The high efficiency of both global and local information flow in the brain or the 'small-world' topology is thought to be important for adapting to environmental demands (Bullmore & Sporns, 2009, 2012). Accordingly, patients with recurrent MDD may be less able to deal with distress from negative life events due to their disrupted intrinsic functional brain topology, making them vulnerable to relapse into depression. The effects of antidepressant medications may also be involved. In a longitudinal study (Li *et al*., 2021), FC was decreased in almost all brain networks after patients were administered escitalopram or duloxetine for 8 weeks. Other factors, such as illness duration, may also contribute. Since most recurrent MDD patients have long histories of medication use, future studies will need to collect more information on the medication usage of patients to better identify medication effects on the properties of functional brain networks of patients with MDD.

### Altered dynamic FC in MDD

Recent studies have highlighted the dynamic aspects of intrinsic brain activity and its role in the pathology of MDD (Demirtas *et al*., 2016; Hou *et al*., 2018; Wang *et al*., 2020). However, most of these studies are preliminary and findings have been inconsistent. Accordingly, a comprehensive study was conducted to characterize altered dynamic FC in MDD at both local and global levels (Long *et al*., 2020; Sizemore & Bassett, 2018). A dynamic network-based framework based on the sliding-window approach was used to estimate several spatio-temporal dynamic network features such as temporal variability, temporal clustering, and temporal efficiency. A total of 460 patients with MDD and 473 HCs were selected for statistical analysis according to their age, education, imaging quality, etc. (for details of selection criteria, please refer to Long *et al*., 2020). Results showed significantly increased temporal variability (*F* = 10.218, *P* = 0.000216, FDR corrected), decreased temporal correlation coefficient ( *F* = 15.071, *P* = 0.0000333, FDR corrected), and shorter characteristic temporal path length ( *F* = 8.768, *P* = 0.000314, FDR corrected) in MDD patients (Fig. 1C). These effects were significant in both first-episode drug naïve (FEDN) and non-FEDN patients. In addition, temporal variability (*ρ* = 0.111, *P* = 0.045) and temporal efficiency (*ρ* = −0.101, *P* = 0.045) were correlated with Hamilton depression rating scale (HAMD) scores after adjusting for age, sex, and site effects in patients with MDD. These results indicate that MDD patients fail to maintain relatively stable brain networks over periods of time and some aberrant connections may interfere with normal interactions among brain regions (Sun *et al*., 2019; Zalesky *et al*., 2014).

### Altered functional lateralization features in MDD

Brain asymmetry has been proposed to be a critical feature of the human brain in both structure and function (Toga & Thompson, 2003). Functional lateralization was characterized with a novel metric, the parameter of asymmetry (PAS) (Ding *et al*., 2021). The PAS was defined as the difference between the mean inter-hemisphere FC and intra-hemisphere FC for a given voxel. We found significantly increased PAS scores in patients with MDD compared with HCs, indicating decreased hemispheric lateralization (Fig. 1D). On the other hand, interhemispheric functional integration is also an important aspect of the brain's functional architecture that can be examined by a voxel-wise measurement called voxel-mirrored homotopic connectivity (VMHC) (Stark *et al*., 2008; Zuo *et al*., 2010). VMHC was compared between 1004 patients with MDD and 898 HCs from the REST-meta-MDD project (Deng *et al*., 2021). Decreased VMHC in MDD was revealed in a wide range of brain regions, including posterior cingulate cortex (PCC), medial prefrontal cortex (MPFC), pre-/post-central gyrus, and inferior frontal and occipital gyrus (Fig. 1E). Such reduced homotopic resting-state FCs may be caused by disrupted structural connectivity such as reduced fractional anisotropy in the corpus callosum (van Velzen *et al*., 2019).

### MDD subgroups

MDD is a highly heterogeneous disorder, probably containing subgroups that correspond to different pathologies and treatments (Drysdale *et al*., 2016). Leveraging the REST-meta-MDD sample, MDD patients were categorized into subgroups according to their resting state FC patterns using a data-driven approach (Liang *et al*., 2020). *K*-means clustering divided MDD patients into two groups depending on their within-DMN FC pattern. One group was characterized by enhanced FCs within the DMN, especially FC between MPFC and PCC, while the other group featured decreased FCs within the DMN (Fig. 1F). These results illustrate a complex pattern of abnormalities in DMN FC, which would be difficult to observe in traditional case-control analyses. Finally, although the REST-meta-MDD project primarily focused on the functional neuropathology associated with MDD, structural alterations in MDD were examined by analyzing the T1-weighted anatomical images collected along with R-fMRI data (Liu *et al*., 2021). Specifically, MDD patients were divided into patients with gastrointestinal symptoms (GI group) and those without GI symptoms (non-GI group). GI symptoms are common in MDD and associated with poorer prognosis (Kop, 2012). Results showed significantly different grey matter volume (GMV) in temporal and occipital regions, thalamus, prefrontal, and postfrontal gyrus among GI, non-GI MDD patients, and HCs (Fig. 1G). The GI group had increased grey matter density in bilateral thalamus compared with the non-GI group. Larger grey matter density in the GI group was also found in right temporal gyrus, fusiform, and lingual gyrus compared with HCs. These results demonstrated that Chinese patients with MDD who experience GI symptoms have abnormalities in grey matter structures.

Once the REST-meta-MDD project entered the unrestricted sharing phase, researchers from outside DIRECT began to conduct additional exploratory analyses. For example, Tozzi and colleagues (2021) re-analyzed within-DMN FCs in terms of the three DMN subsystems (Andrews-Hanna, 2012; Andrews-Hanna *et al*., 2010). Recent empirical evidence showed that the DMN can be fractionated into three subsystems: a core subsystem that corresponds to self-referential thinking (the core subsystem); a subsystem that is anchored in the dorsal PFC and corresponds to cognition related processes; and a subsystem that is anchored in the medial temporal lobe (the MTL subsystem) and corresponds to autobiographical memory (Andrews-Hanna, 2012; Andrews-Hanna *et al*., 2010). They found that only FC within the core subsystem was significantly reduced in MDD compared to NCs. These results have expanded the research scope of REST-meta-MDD and show the potential of this rich repository of clinical data.

Box 1. **Directions for future DIRECT research**

1. What are the differences regarding MDD abnormalities in different ethnic groups (e.g. Chinese vs. Caucasian)? What factors contribute such differences (e.g. response styles, thinking styles or genetic factors?)
2. To what extent can it help improve the reproducibility of results to transfer preprocessing pipelines from volume-based approaches [i.e. DPSRSF (Yan & Zang, 2010) and SPM (Ashburner, 2012)] to surface-based approaches [e.g. DPABISurf (Yan *et al*., 2021) and fMRIPrep (Esteban *et al*., 2019)]?
3. What are the differences and similarities among the neuroimaging alterations across different mental disorders?
4. What are the longitudinal effects of antidepressant medications on the brain?
5. What are the white matter alterations in MDD?
6. Can we guide neuromodulation techniques (e.g. TMS) through brain network mechanisms we identified with fMRI?

## Future Directions for the DIRECT Consortium

In this review, we have briefly described the motivation and evolution of the DIRECT consortium and the main published findings based on its first project, REST-meta-MDD. Through this endeavour, we demonstrated that pooling R-fMRI data across multiple sites with standardized processing protocols can substantially boost statistical power and detect subtle but reliable MDD-related abnormalities in brain. Furthermore, we established an open-access data repository to make all shared functional data available to the broad scientific community. We hope this will advance discovery-based analyses seeking neuroimaging biomarkers, a deeper understanding of MDD's neuropathology, and development of novel treatments for MDD. Despite the inspiring and unique findings emerging from the present research based on REST-meta-MDD and DIRECT, further questions can be raised. An important limitation is the exclusively Chinese sample. Thus, one critical next step of the DIRECT consortium is to extend the Chinese sample to other ethnic groups such as Caucasians through international collaborations. Other directions include: (i) improving reproducibility and sensitivity by using a surface-based, state-of-the-art pipeline (DPABISurf) (Yan *et al*., 2021); (ii) accumulating functional neuroimaging data from other psychiatric disorders such as bipolar disorder and schizophrenia; (iii) longitudinal research targeting the effects of antidepressant medications on brain networks in MDD; (iv) quantifying alterations in structural connections in MDD with diffusion tensor imaging (DTI); and (v) novel individualized neuromodulation approaches (e.g. transcranial magnetic stimulation, TMS) with fMRI-based anatomic targeting.

### Identifying effects of different cultural groups

MDD in different ethnic groups has been reported to have different prevalence rates, heterogeneous subtypes, and varied treatment outcomes (Budhwani *et al*., 2015; Lee *et al*., 2014; Lesser *et al*., 2007). As this planet's largest ethnic group, the Chinese have been reported to exhibit lower rates of depression (Huang *et al*., 2019; Parker *et al*., 2007; Parker *et al*., 2001). The reasons for this are the focus of continuing debate. Some claim that the Chinese tend to express depression somatically and deny feelings of distress (Qiu *et al*., 2018). From the cultural viewpoint, some argue that Chinese beliefs and ways of responding to emotions (i.e. holistic thinking styles) make Chinese people less vulnerable to the negative affects of distress (De Vaus *et al*., 2018). Genetic factors may also contribute. The s/s allele of serotonin transporter is more prevalent in East Asians (45–74%) compared to Caucasians (12–24%) (Goldman *et al*., 2010). Furthermore, the s/s genotype is associated with a higher risk of MDD in Caucasians but not in Asians (Kiyohara & Yoshimasu, 2010). Thus, results from an exclusive Chinese sample may not generalize to other ethnic groups. In 2012, the international Enhancing NeuroImaging Genetics through Meta-Analysis (ENIGMA) initiative launched its MDD consortium, to identify neuroimaging alterations associated with MDD and their modulators (Schmaal *et al*., 2020). So far, neuroimaging data from more than 9000 HCs and 4000 MDDs have been accumulated by ENIGMA MDD consortium. Initial attempts have been made to pool data from DIRECT and ENIGMA MDD to identify the potential ethnic and cultural factors (e.g. directly compare the differences regarding whole-brain FC maps between Caucasian and Chinese MDD patients) in the neuropathology of MDD. We believe direct comparison across a large cross-cultural sample can provide unprecedentedly powered evidence for the contribution of ethnic factors to the structural and functional alterations associated with MDD.

### Towards surface-based analyses

Obtaining more reliable and reproducible findings from functional neuroimaging data has become a major challenge for the field (Botvinik-Nezer *et al*., 2020; Chen *et al*., 2018). Preprocessing of R-fMRI data is complex and contains numerous steps to yield clean data for further statistical analyses. Outdated and flexible *ad hoc* preprocessing pipelines have been shown to decrease the quality and consistency of results (Esteban *et al*., 2019). In the REST-meta-MDD project, this issue was addressed by conducting a standardized, volume-based preprocessing pipeline based on DPARSF. However, recent methodological benchmarks have highlighted the drawbacks of volume-based preprocessing approaches, calling for a transformation to surface-based approaches (Coalson *et al*., 2018; Zuo *et al*., 2013). One obstacle in applicating state-of-the-art surface-based approaches in a multi-site consortium like DIRECT is the lack of a 'turn-key' toolbox. Accordingly, the DPABISurf pipeline was developed with a user-friendly graphical user interface that requires no scripting skills from users (Yan *et al*., 2021). DPABISurf is the latest upgrade of the widely used preprocessing pipeline DPARSF/DPABI (Yan *et al*., 2016; Yan & Zang, 2010) and follows the same designing concept. On the basis of this pipeline, future pooling of preprocessed time series in DIRECT will be produced with a surface-based approach, which should enhance the reliability and sensitivity of future DIRECT studies.

### Transdiagnostic investigation

Evidence from ENIGMA has implicated a shared structural abnormality pattern among MDD, bipolar disorder, and schizophrenia (Schmaal *et al*., 2020). Genomewide association studies have also suggested that implicated genes may have pleiotropic effects across disorders (Huang *et al*., 2010). Furthermore, the differential diagnosis of bipolar disorder and MDD has long been a challenge for clinicians, indicating an overlap of clinical presentation between these disorders (Hirschfeld, 2014). Thus, investigating and characterizing shared and unique functional/structural brain alterations across these disorders may be particularly important and can help develop image-based diagnostic biomarkers to assist differential diagnosis. DIRECT is building a transdiagnostic dataset together with new participating research groups. Preliminary analyses to explore similarities as well as differences among these disorders regarding brain function and structure are anticipated.

### Identifying longitudinal effects of antidepressant medications

One contribution of a pooled large-scale R-fMRI data repository is to generate hypothesis for future longitudinal studies. Effect sizes of studies using a within-subject design are larger than those using a cross-sectional between-subject design (Chen *et al*., 2018). However, longitudinal studies require more resources and a targeted design, so a sufficient prior knowledge base is needed to narrow the exploration scope. The present DIRECT studies have highlighted the effects of antidepressant medications on MDD patients’ functional brain networks, especially the DMN. To test this, the effects of antidepressant treatment were studied in a group of 41 first-episode drug-naïve patients with MDD who were administrated escitalopram or duloxetine for 8 weeks (Li *et al*., 2021). FCs within and among brain networks were generally decreased after antidepressant treatment, confirming the findings from the original DIRECT studies. The longitudinal effects of antidepressant medications on large-scale brain networks will be the focus of a future study that is being planned.

### Exploring white matter alterations in MDD

DTI is an effective *in vivo* technique to investigate white matter microstructural properties of psychiatric patients (Rae *et al*., 2012). Multi-site studies have been conducted to characterize MDD patients’ white matter abnormalities both cross-sectionally (van Velzen *et al*., 2019) and longitudinally (Davis *et al*., 2019). It can be challenging to apply sophisticated and standardized workflows across many sites, so existing large-scaled multi-site studies tend to concentrate on more straightforward metrics such as fractional anisotropy. Recent advances in building an integrative software platform for DTI preprocessing (e.g. QSIPrep: Cieslak *et al*., 2021) have made it possible to pool prepossessed DTI images and conduct more advanced analyses such as tractography and mapping the structural connectome. Building a DTI repository under the framework of DIRECT to determine abnormalities regarding structural connection properties in MDD is under discussion.

### Developing network-targeted neuromodulation therapy

Neuromodulation techniques, especially TMS, have the potential to treat MDD (Lefaucheur *et al*., 2020). Initial findings from DIRECT highlight the critical role the DMN plays in the neurophysiology of MDD. DMN abnormalities in MDD have long been associated with rumination, a passive and repetitive thinking style that is common in MDD patients (Hamilton *et al*., 2015; Kaiser *et al*., 2015). A recent hypothesis-driven study found that FCs between the core and MTL subsystems were enhanced during rumination, while FCs between core and DMPFC subsystems were reduced (Chen *et al*., 2020). Further analyses showed that the dynamic stability of the DMN was also decreased during rumination (Chen & Yan, 2021). These findings indicate that it might be possible to inhibit rumination by directly modulating DMN FC patterns through novel neuromodulation approaches such as TMS. Current TMS approaches show promising antidepressant effects, but effect sizes are modest and treatment duration is long (Lefaucheur *et al*., 2020). Transforming present scalp-based targeting to individualized fMRI guided targeting may improve the efficiency of TMS (Cash *et al*., 2020). Indeed, one recent double-blinded randomized controlled trial (Cole *et al*., 2021) found that targeting an individualized left dorsal lateral prefrontal cortex (DLPFC) region that is anticorrelated to subgenual anterior cingulate cortex was highly effective (remission rate 79%), indicating the feasibility of generating individualized targets for TMS in relation to specific brain networks. Future DIRECT research intends to develop target searching algorithms according to the subsystem mechanisms underlying rumination and set up a clinical trial to test the antidepressant effects of such neuromodulation therapy.

## Conclusion

In sum, the DIRECT consortium has accumulated an unprecedently large functional neuroimaging repository by initiating the REST-meta-MDD project. Studies based on this dataset have provided highly powered evidence for the field of neuropathology of MDD that has been beset by contradictory results. Furthermore, some intriguing insights have emerged from initial analyses. The second stage of data sharing under the framework of DIRECT is underway and several longitudinal studies based on hypotheses from REST-meta-MDD have been launched. We hope these endeavours will advance the translation of neuroimaging studies to clinical practice.

## Notes

DIRECT consortium: Chao-Gan Yan, Ph.D.^1,2,3,4,5,6^, Xiao Chen, Ph.D.^1,2,3,4^, Li-Ping Cao, M.D.^9^, Wei Chen, M.D.^11^, Yu-Qi Cheng, M.D.^12^, Yi-Ru Fang, M.D.^13^, Qi-Yong Gong, M.D.^14,15^, Wen-Bin Guo, M.D.^16^, Li Kuang, M.D.^17^, Bao-Juan Li, M.D.^18^, Tao Li, M.D.^20,21^, Yan-Song Liu, M.D.^22^, Zhe-Ning Liu, M.D.^16^, Jian-Ping Lu, M.D.^23^, Qing-Hua Luo, M.D.^17^, Hua-Qing Meng, M.D.^17^, Dai-Hui Peng, M.D.^13^, Jiang Qiu, Ph.D.^24^, Yue-Di Shen, M.D.^25^, Tian-Mei Si, M.D.^26^, Yan-Qing Tang, M.D.^27^, Chuan-Yue Wang, M.D.^28^, Fei Wang, M.D.^27,29^, Hua-Ning Wang, M.D.^18^, Kai Wang, M.D.^30^, Xiang Wang, M.D.^16^, Ying Wang, M.D.^31^, Xiao-Ping Wu, M.D.^32^, Chun-Ming Xie, M.D.^33^, Guang-Rong Xie, M.D.^16^, Peng Xie, M.D.^34,35,36^, Xiu-Feng Xu, M.D.^12^, Hong Yang, M.D.^37^, Jian Yang, M.D.^35^, Shu-Qiao Yao, M.D.^16^, Yong-Qiang Yu, M.D.^38^, Yong-Gui Yuan, M.D.^39^, Ke-Rang Zhang, M.D.^40^, Wei Zhang, M.D.^41^, Zhi-Jun Zhang, M.D.^33^, Jun-Juan Zhu, M.D.^42^, Xi-Nian Zuo, Ph.D.^43,44^, Jing-Ping Zhao, M.D.^16^, Yu-Feng Zang, M.D.^45,46^
